# Importance of the cytomegalovirus seropositive recipient as a contributor to disease burden after solid organ transplantation

**DOI:** 10.1016/j.jcv.2012.02.020

**Published:** 2012-06

**Authors:** Vincent C. Emery, Kevin Asher, Cristina de Juan Sanjuan

**Affiliations:** a Department of Infection (Royal Free Campus), University College Medical School, Rowland Hill Street, Hampstead, London NW3 2QG, UK; b Roche Products Limited, 6 Falcon Way, Welwyn Garden City AL7 1TW, UK

**Keywords:** CMV, cytomegalovirus, D, donor, R, recipient, Audit, Immunocompromised host, Transplantation

## Abstract

**Background:**

The incidence of cytomegalovirus (CMV) syndrome/disease after adult solid organ transplantation in the era effective antiviral therapy has not been fully assessed.

**Objective:**

To determines the incidence of CMV syndrome/disease after solid organ transplantation in the UK.

**Study design:**

A retrospective analysis of 1807 solid organ transplants from 12 UK solid organ transplant centres representing 32.7% of all transplant activity occurring in the UK between 1/04/2004 and 31/03/2006. Patients were categorised into those experiencing an episode of symptomatic CMV infection after transplant or those who remained free of symptoms. All patients were followed up for 2 years for the occurrence of CMV syndrome/disease.

**Results:**

The majority of the transplant centres used valganciclovir prophylaxis in the high risk D+R− patients (91.6%) whereas management of the lower risk D+R+ and D−R+ patients was more variable with deployment of both prophylactic and pre-emptive strategies in ∼50% of centres. CMV syndrome/disease occurred in 20.5% of the D+R− patients representing 55 cases whereas the incidence was only 8.1% and 9% in the D+R+ and D−R+ group, respectively (*p* < 0.001 compared to the D+R− group), but representing a further 58 cases of CMV syndrome/disease. CMV viraemia in the D+R− group was associated with a high probability (65%) of CMV syndrome/disease in renal transplant recipients whereas this was less apparent in the intermediate risk groups.

**Conclusions:**

CMV syndrome/disease remains an important healthcare burden after solid organ transplantation with the intermediate risk groups contributing similar numbers of cases as the high risk group.

## 1 Background

Historically cytomegalovirus (CMV) disease was associated with a high morbidity and mortality after solid organ transplantation.^1^ In recent years, the combination of improved antiviral management and immunosuppressive strategies has minimised the health impact of CMV in this clinical setting.^2–6^ Despite the widespread deployment of antiviral prophylaxis for high-risk patients reducing the risk of infection and disease during the prophylaxis period late infection and disease remain important clinical management challenges.^7–10^ In addition, the appropriate antiviral management (prophylaxis or pre-emptive antiviral therapy) of patients at intermediate risk of infection and disease remains controversial even though these patients represent a sizeable transplanted population.^11^

While clinical trials provide essential benchmarks for drug efficacy there remains an important place for information gathered from audits across multiple centres to inform healthcare managers and physicians on the current impact that CMV has following solid organ transplantation.

## 2 Objectives

To assess the incidence of CMV syndrome/disease after solid organ transplantation especially in the recipient CMV seropositive population.

## 3 Study design

The survey was a retrospective analysis of centres that transplanted solid organs within the UK between 1/04/2004 and 31/03/2006. Patients were categorised into either those who developed an episode of symptomatic CMV infection after transplant or those who remained free of symptoms related to CMV. All patients were stratified according to their risk of CMV infection based upon donor and recipient CMV serology. Patients were followed up for 2 years for the occurrence of CMV syndrome/disease (defined below). However, patients were excluded from the final analysis if their care transferred to another centre within 2 years of follow-up.

### 3.1 Primary objective

The primary objective of the study was to characterise the frequency of symptomatic CMV infection after solid organ transplantation in the UK setting.

### 3.2 Inclusion/exclusion criteria

Patients who received a solid organ transplant within the UK between 1/04/2004 and 31/03/2006. There were no exclusion criteria.

### 3.3 Participating centres

All UK hospitals performing adult heart, lung and liver transplantation were approached to participate in the study along with 2 centres performing kidney transplantation only. This represented 21 of 36 adult transplanting units. Nine hospitals representing 12 transplant units participated in the study. Information on the antiviral CMV management strategy in place during the study period was collected from each centre. The study was conducted in accordance with good clinical practice (GCP) guidelines.

### 3.4 CMV disease definitions

CMV syndrome was defined as CMV PCR (polymerase chain reaction) viraemia plus fever of unexplained origin and one of the following signs: leucopenia, myalgia or arthralgia.^11^

CMV disease was defined according to the Ljungman et al.:^12^

(1) Detection of CMV by culture, histopathology, immunohistochemistry with CMV specific antibodies or in situ hybridisation in a biopsy of the affected organ.
(2) CMV central nervous system (CNS) disease could be diagnosed by the presence of CMV DNA or virus culture positivity in the cerebrospinal fluid.
(3) CMV retinitis diagnosed by qualified ophthalmologist.
(4) CMV hepatitis diagnosed by the presence of CMV in a liver biopsy by histology (CMV inclusions or immunohistochemistry).
(5) CMV colitis diagnosed by the presence of CMV in a gut biopsy by histology (CMV inclusions or immunohistochemistry).

Patients experiencing CMV viraemia without disease were classified as having asymptomatic viraemia. No data on viral load for CMV were obtained as part of the audit. In centres adopting pre-emptive therapy, CMV surveillance was undertaken by real time PCR methods (either commercial or in-house on plasma or whole blood) on a weekly basis until 3-months post transplant. Consistent post prophylaxis surveillance was not routinely adopted. Thresholds for initiation of therapy varied between centres but first-line therapy involved either intravenous ganciclovir (5 mg/kg bid) or valganciclovir (900 mg bid) and was adjusted for renal function.

### 3.5 Statistical analysis

Comparison of incidence between groups was performed using the chi-squared test or Fisher's exact test as appropriate. *p*-values ≤0.05 were regarded as significant.

## 4 Results

Nine hospitals participated in the study performing 1807 solid organ transplants during the 2-year study period. The majority were renal transplant recipients (57.3%) with the next largest population being liver transplant recipients (28.6%). The total number of solid organ transplants carried out in the UK during the study period was 5518 (UK Transplant Registry data) and so the participating centres in our study represented 32.7% of all transplant activity occurring in the UK during the 2 year period from 2004 to 2006. The distribution of transplants amongst the 9 hospitals is shown in Table 1.

The individual antiviral management strategies adopted for each solid organ transplant group stratified according to the donor and recipient serostatus for CMV is shown in Table 2. In the high-risk D+R− group the majority of CMV protocols deployed antiviral prophylaxis with valganciclovir (VGCV) at 900 mg once a day for 90 days (76.4%) with the remaining protocols used a lower dose of VGCV (450 mg od for 90 days) and one heart transplant protocol deployed no prophylaxis or pre-emptive therapy in this high risk group. In the intermediate risk D+R+ group, a more diverse range of management strategies were deployed with 47% of centres performing neither prophylaxis nor pre-emptive therapy, 35% performing prophylaxis with VGCV at either 900 mg once daily or 450 mg once daily for 90 days and 17% performing pre-emptive therapy with valganciclovir at full dose. In the low risk category (D−R+), the majority of transplant units (53%) were performing neither prophylaxis nor pre-emptive therapy. With a single exception, the remaining centres followed a management strategy for the D−R+ patients that was the same as for the D+R+ group. For patients with virtually no risk of CMV infection and disease after transplantation (donor and recipient both seronegative, D−R−), the majority of CMV protocols (82.3%) undertook no antiviral prophylaxis or pre-emptive therapy. However, 3 centres adopted pre-emptive therapy for these low risk patients.

The incidence of CMV disease in the different patient populations who had achieved 2 years of follow-up stratified according to donor and recipient seropositivity is shown in Table 3. CMV disease continues to be most prevalent in the high risk D+R− setting (despite the fact that majority of these patients were receiving prophylaxis) with an average rate of 20.5%. The rate was reduced significantly in the D+R+ group (8.1%; *p* < 0.001 vs D+R−) but it was noteworthy that the prevalence in the lung transplant recipients was comparable to that observed in the high-risk population. The D+R+ renal transplant recipients had a 40% reduction in their incidence of CMV disease compared to the D+R− group. In the D−R+ group, the average incidence of CMV disease was lower than that observed in the D+R− group (mean 9.0%; *p* < 0.001) and comparable to that observed in the D+R+ group although the high incidence of disease in both lung and heart transplant recipients should be noted. Overall the number of patients with CMV syndrome/disease in the recipient seropositive group was comparable to the number of cases of syndrome/disease in the D+R− group (58 cases in the R+ group vs 55 cases in the D+R− group; *p* = not significant). The incidence of overt CMV disease in the D−R− group was very low (0.1%) equating to a single liver transplant recipient.

In the context of CMV viraemia and its association with the occurrence of CMV syndrome and disease, data were available for 4 renal transplant centres and 3 liver transplant centres representing a total of 1547 patients (85.6% of the total patient evaluable). These data are summarised in Tables 4 and 5, respectively. In the D+R− renal transplant recipients the overall rate of CMV viraemia was 36.2% with the rate of asymtomatic CMV viraemia relatively low at 12.1% whereas CMV syndrome with concurrent viraemia was recorded at a higher level (22.4%) and overt CMV disease only observed in one patient. In contrast, both the D+R+ and the D−R+ renal transplant groups exhibited higher levels of asymptomatic CMV viraemia (24.1% and 32.8%, respectively) compared to the D+R− group although the incidence of CMV syndrome was significantly lower in both groups compared to the high risk group (*p* = 0.026 for D+R+ and *p* = 0.0012 for the D−R+ comparison). In patients where both donor and recipient were seronegative the incidence of asymptomatic viraemia and syndrome were extremely low (Table 4).

In a similar analysis of liver transplant recipients, the overall incidence of asymptomatic CMV viraemia was lower than that observed across all donor–recipient CMV groups after renal transplantation although it only reached statistical significance for the D−R+ comparison (*p* = 0.003). Consistent with the data in the high risk D+R− renal transplant group there was a lower rate of asymptomatic CMV viraemia (7.7%) compared to the occurrence of CMV syndrome/disease (21.3%). This effect was not observed in the other donor/recipient groups where the incidence of asymptomatic viraemia was always greater than CMV syndrome/disease (Table 5).

We next investigated whether the management strategy adopted in the lower risk (D+R+ and D−R+) settings was associated with differences in CMV syndrome/disease incidence. In seropositive renal transplant recipients receiving a seropositive donor organ, CMV syndrome/disease was reduced by approximately 43% (10/63 to 6/65; *p* = 0.11) by the deployment of pre-emptive therapy compared to the no therapy group while in the low risk D−R+ setting a 62% reduction (3/46 to 1/40 *p* = 0.8) in incidence of CMV syndrome/disease was achieved through pre-emptive therapy. A similar comparison for pre-emptive therapy in liver transplant recipients could not be carried out but in the D+R+ group, receipt of CMV prophylaxis was associated with a 62.5% reduction (6/75 to 2/66; *p* = 0.28) in CMV syndrome/disease compared with no prophylaxis. Combining both patient groups revealed that any antiviral intervention reduced the incidence of CMV syndrome/disease by 58% (*p* = 0.033).

## 5 Discussion

Historically, CMV syndrome and disease have had a major impact on patient wellbeing after transplantation.^1^ However, very few studies have attempted to quantify the impact that recent advances in clinical management has had on the incidence of CMV syndrome and disease outside of formal clinical trials. The results of the present audit show that CMV syndrome/disease remains an important and prevalent problem following solid organ transplantation in the UK. In the high risk D+R− group, the average incidence of CMV syndrome/disease was 20.5% consistent with previous data for renal and liver transplant recipients receiving 90 days of valganciclovir prophylaxis during clinical trials.^7–10^ There appears to be very few viral or immunological markers that accurately predict the subset of patients who will develop CMV syndrome/disease after prophylaxis^13,14^ although one study has observed that T-cell responses at day 100 were predictive of protection against symptomatic infection.^15^ In the current study we observed that 75% and 65% of high risk renal and liver transplant recipients, respectively with CMV viraemia experienced CMV syndrome/disease arguing that surveillance of CMV viraemia following prophylaxis remains an important diagnostic. The utility of such an approach will depend upon the sampling frequency of CMV viraemia after the cessation of prophylaxis^16,17^ as other studies have indicated that CMV viraemia post prophylaxis is not a good surrogate for impending disease.^18,19^ The majority of centres still use prophylaxis for CMV in high risk patients so we believe that our audit data is an accurate reflection of current practice with the caveat that some renal transplant centres may have moved to 200 days of prophylaxis based upon the results of the recent IMPACT study.^8^

Although the high-risk D+R− solid organ group is a major concern for the transplant team, the incidence of symptomatic infection in the lower risk strata should not be ignored.^11,20^ In this audit, D+R+ patients had an average incidence of CMV syndrome/disease of 8.1% although there was significant variation within the transplant groups with the lung transplant group having an incidence that was comparable with the D+R− group. Even in the D−R+ setting, CMV syndrome/disease was observed in 9.0% of patients and was particularly prevalent in recipients of heart and lung transplants. Such observations argue that correct management strategies for these intermediate risk patients need to be in place since they accounted for 58 cases of disease (51.3%) during the study period which was comparable to 55 cases observed in the D+R− group. It was also noteworthy that in the renal transplant setting pre-emptive therapy substantially reduced syndrome/disease compared to no therapy and in the liver transplant setting prophylaxis of the D+R+ group with VGCV for 90 days had a similar effect compared to no treatment. Although this audit comprised patients who were transplanted 7 years ago there is little evidence that prophylaxis has increased dramatically in this intermediate risk group possibly reflecting the lack of controlled clinical trials in this group. In addition, the incidence of CMV viraemia in patients being managed pre-emptively or undergoing no active management was comparable arguing many patients who went on to suffer early signs and symptoms of CMV were in fact assessed for CMV viraemia.

There was only one case of CMV disease in the D−R− group. This group remains at very low risk of symptomatic CMV infection and based on our data we would argue that this group requires no active antiviral management outside of general clinical awareness of the symptoms of primary CMV infection.

In conclusion, this audit indicates that, in the UK, CMV syndrome/disease remains a frequent occurrence following solid organ transplantation and centres should endeavour to ensure that management strategies across all risk groups are robust and audited regularly to minimise the impact of CMV on quality of life and ultimately graft survival.

## Funding

This audit was coordinated and funded by Roche Products Limited. Work in the laboratory of VCE is supported by the UK Medical Research Council through a Centre grant for Medical Molecular Virology and by the Wellcome Trust.

## Competing interests

Professor Vincent Emery has received honoraria for advisory boards and talks for Roche Products Ltd. Kevin Asher and Cristina de Juan Sanjuan were employees of Roche Products Ltd. at the time of the study.

## Ethical approval

This audit was approved by the Royal Free Hospital & Medical School Research Ethics Committee reference 08/H0720/83.

## Contributors

KA, VCE and CdJS were involved in the audit design and appropriate ethical submissions. Statistical analysis was carried out by CdJS. VCE wrote the paper with input and review from KA and CdJS.

## Figures and Tables

**Table 1:** Distribution of patients across the 9 participating UK centres.

Hospital ID	Kidney	Liver	Pancreas	Heart	Lung
A	155	112	13^a^		
B	93	94	26^b^		
C				70	51^c^
D		67			
E	159		28^d^		
F	216	244			
G	99				
H	313				
I				32	35

Total	1035	517	67	102	86

a All simultaneous kidney-pancreas (SKP).

b 23 SKP + 3 pancreas only.

c 6 heart/lung + 11 single lung + 34 bi-lateral lung.

d 26 SKP + 2 pancreas only.

**Table 2:** CMV antiviral management strategies deployed in different solid organ transplant recipients stratified according to CMV donor and recipient serostatus.

Organ	Number of centres	D+R−	D+R+	D−R+	D−R−
Kidney	3	VGCV 900 mg 90 d	None	None	None
	2	VGCV 900 mg 90 d	PET	PET	PET
	1	VGCV 900 mg 90 d	VGCV 900 mg 90 d^a^	VGCV 900 mg 90 d^a^	None

Liver	3	VGCV 900 mg 90 d	None	None	None
	1	VGCV 900 mg 90 d	VGCV 900 mg 90 d	None	None

SPK/Pancreas	1	VGCV 450 mg 90 d	VGCV 900 mg 90 d	VGCV 900 mg 90 d	None
	1	VGCV 900 mg 90 d	PET	PET	PET
	1	VGCV 900 mg 90 d	VGCV 900 mg 90 d	VGCV 900 mg 90 d	None

Heart	1	VGCV 450 mg 90 d	VGCV 450 mg 90 d	VGCV 450 mg 90 d	None
	1	None	None	None	None

Lung	1	VGCV 450 mg 90 d	VGCV 450 mg 90 d	VGCV 450 mg 90 d	None
	1	VGCV 900 mg 90 d	None	None	None

*Key*: SPK = simultaneous pancreas and kidney and (includes 1 pancreas only transplant); VGCV = valganciclovir; 900 mg = daily prophylactic dose for normal renal function; PET = pre-emptive therapy; 90 d = 90 days

a Only if patient has received Campath therapy.

**Table 3:** Incidence of CMV disease (including syndrome) in different organ transplant recipients according to their donor and recipient serostatus for CMV for patients with 2 year follow-up data, who lost their graft or died during the 2 year period.

	D+R−		D+R+		D−R+		D−R−		Unknown	
Transplant group	*N*	CMV disease (%)	*N*	CMV disease (%)	*N*	CMV disease (%)	*N*	CMV disease (%)	*N*	CMV disease (%)
Renal	143	21.5	203	12.8	144	4.9	178	0.6	56	3.6
Liver	73	20.5	141	5.7	142	2.8	104	0.0	19	0.0
SPK	12	25.0	1	0.0	11	0.0	18	0.0	3	33.3
Heart	33	6.1	16	0.0	22	18.2	31	0.0	0	0.0
Lung(s)	17	29.4	18	22.2	26	19.2	19	0.0	6	0.0

Totals	278	20.5	379	8.1	345	9.0	350	0.1	84	7.4

*Key*: SPK = simultaneous pancreas and kidney and (includes 1 pancreas only transplant).

**Table 4:** Incidence of asymptomatic CMV viraemia in renal transplant patients and its relationship with CMV syndrome/disease in a subset of 4 transplant centres.

	D+R−a		D+R+^b^		D−R+^b^		D−R−	
	*N*	Incidence (%)	*N*	Incidence (%)	*N*	Incidence (%)	*N*	Incidence (%)
No CMV viraemia	37	63.8	72	66.7	42	65.6	78	96.3
Asymptomatic viraemia	7	12.1	26	24.1	21	32.8	2	2.5
CMV syndrome	13	22.4	8	7.4	1	1.6	1	1.2
CMV disease	1	1.7	2	1.9	0	0.0	0	0.0

a All patients received VGCV at 900 mg od (adjusted for renal function) for 90 days.

b 2 centres used pre-emptive therapy for CMV management.

**Table 5:** Incidence of asymptomatic CMV viraemia in liver transplant patients and its relationship with CMV syndrome/disease in a subset of 3 transplant centres.

Parameter	D+R−a		D+R+^b^		D+R+^c^		D−R+	
	*N*	Incidence (%)	*N*	Incidence (%)	*N*	Incidence (%)	*N*	Incidence (%)
No CMV viraemia	37	71.2	38	79.2	48	92.3	93	94.0
Asymptomatic viraemia	4	7.7	6	12.5	3	5.8	3	3.0
CMV syndrome	7	13.5	4	8.3	1	1.9	2	2.0
CMV disease	4	7.7	0	0.0	0	0.0	1	1.0

a All 3 units used VGCV at 900 mg od (adjusted for renal function) for 90 days.

b 2 units used no proactive CMV management.

c Single unit using VGCV 900 mg od (adjusted for renal function).
